# EZH2 inhibits autophagic cell death of aortic vascular smooth muscle cells to affect aortic dissection

**DOI:** 10.1038/s41419-017-0213-2

**Published:** 2018-02-07

**Authors:** Rui Li, Xin Yi, Xiang Wei, Bo Huo, Xian Guo, Cai Cheng, Ze-Min Fang, Jing Wang, Xin Feng, Ping Zheng, Yun-Shu Su, Jackson Ferdinand Masau, Xue-Hai Zhu, Ding-Sheng Jiang

**Affiliations:** 1 Division of Cardiothoracic and Vascular Surgery, Tongji Hospital, Tongji Medical College, Huazhong University of Science and Technology, Wuhan, 430030 China; 2 Department of Cardiology, Renmin Hospital of Wuhan University, Wuhan, 430060 China; 3 Cardiovascular Research Institute, Wuhan University, Wuhan, 430060 China; 4 Hubei Key Laboratory of Cardiology, Wuhan, 430060 China; 5 Key Laboratory of Organ Transplantation, Ministry of Education, Tongji Hospital, Tongji Medical College, Huazhong University of Science and Technology, Wuhan, 430030 China; 6 Key Laboratory of Organ Transplantation, Ministry of Health, Tongji Hospital, Tongji Medical College, Huazhong University of Science and Technology, Wuhan, 430030 China

## Abstract

Enhancer of zeste homolog 2 (EZH2), a methyltransferase that di- and tri-methylates lysine-27 of histone H3, largely functions as a transcriptional repressor, and plays a critical role in various kinds of cancers. Here we report a novel function of EZH2 in regulating autophagic cell death (ACD) of vascular smooth muscle cells (VSMCs) that affect aortic dissection (AD). Inhibition of EZH2 activity by UNC1999 or knockdown EZH2 resulted in VSMC loss, while overexpression of EZH2 facilitated VSMC growth, and these effects of EZH2 on VSMCs were independent of proliferation and apoptosis. Interestingly, more autophagic vacuoles and increased LC3II protein levels were identified in VSMCs with EZH2 inhibition or deficiency. Moreover, when compared with counterparts, chloroquine alone, or chloroquine with rapamycin treatment led to more LC3II accumulation in EZH2 inhibited or knockdown VSMCs, which indicated that EZH2 negatively regulated autophagosome formation. In conjunction to this, ATG5 and ATG7 protein levels were remarkably increased in EZH2 inhibited or deficient VSMCs, and ATG5 or ATG7 knockdown virtually rescued VSMC loss induced by EZH2 inhibition or knockdown. In addition, we found that the MEK–ERK1/2 signaling pathway, but not AMPKα, mTOR, or AKT pathway, is responsible for the impact of EZH2 on ACD of VSMCs. Additionally, the adverse effects of EZH2 inhibition or knockdown on VSMCs were largely reversed by PD98059, an inhibitor of MEK1. More importantly, decreased EZH2 expression levels in the aortic wall of patients with AD indicated its contribution to VSMC loss and AD occurrence. Overall, these findings revealed that EZH2 affects ACD of VSMCs and the pathologic process of AD via regulating ATG5 and ATG7 expression and MEK–ERK1/2 signaling. Our hitherto unrecognized findings indicate that EZH2 activation has therapeutic or preventive potential for AD.

## Introduction

According to the 2014 ESC guidelines of aortic diseases, the prevalence of aortic dissection (AD) is around six cases per hundred thousand individuals per year, and of that, 50% of the patients presenting with acute type A AD (TAAD) end up dying within the first 48 h if not operated^1^. The typical morphological feature of aortic wall is medial degeneration in AD patients, including fragmentation and loss of elastic fibers, vascular smooth muscle cell (VSMC) loss, and accumulation of mucopolysaccharides^2–4^. Proliferation inhibition, apoptosis, necrosis, and autophagy enhancement are all possible causes of VSMC loss in the aortic wall^5–8^. The autophagy of VSMCs in the aortic wall was recently identified^5,6^, but the regulatory mechanisms still remain largely unknown.

Autophagy is a cellular self-digestion pathway involved in protein and organelle degradation associated with the formation of autophagosome and the cytosolic double-membrane vesicles that engulf cellular components^9^. Autophagosome formation is regulated by serial activation of protein complexes. The ULK1 complex is responsible for autophagy induction, the class III phosphatidylinositol (PtdIns) 13-kinase-BECN1 complex controls the autophagosome nucleation, and finally the Atg12-ATG5 and the LC3I/LC3-phosphatidy, lethanolamine (PE, LC3II) complexes participate in extension and closure of the autophagosome membranes^10^. Several signaling pathways were reported to regulate autophagy in mammalian cells, especially mTOR, AMPKα, Akt, and MAPK signaling. Although proper autophagy is primarily a protective process for the cell, uncontrolled autophagy activation will lead to cell death, which is defined as “autophagic cell death (ACD)”, also known as “Type II programmed cell death”^10^. However, the mechanisms that control autophagy and whether they are protective or detrimental on cells are largely unknown.

A growing number of studies have demonstrated that histone methyltransferases play an important role in autophagy^11–13^. For example, the histone H3 lysine 9 (H3K9) methyltransferase G9A inhibits cell death with autophagy in various cancer cell lines, while its inhibitors (BRD4770 and BIX01294) induce autophagy^11,14^. Histone methyltransferase enhancer of zester homolog 2 (EZH2), which di- and tri-methylated H3 at lys27 (H3K27me2 and H3K27me3) to suppress gene transcription, is the enzymatically active subunit of polycomb repressive complex (PRC) 2^15^. Previous researches have demonstrated that EZH2 plays a critical role in the pathophysiologic processes of vasculature^15–17^. It maintains the integrity of the developing vasculature via inhibition of Creb3l1, Fosl1, Klf5, and Mmp9 expression^16^. Aljubran et al.^17^ demonstrated that EZH2 is also able to promote the migration and proliferation of pulmonary arterial SMCs. Furthermore, in a limb ischemic mouse model, Mitić et al.^15^ demonstrated that inhibition of EZH2 by DZNep increases angiogenesis in ischemic tissue. However, whether EZH2 plays a role in VSMC loss during pathology process of AD, and whether this effect of EZH2 is related to autophagy, has not yet been determined.

In this study, we demonstrate that the VSMC growth is inhibited by EZH2 inhibition or knockdown, while being promoted by EZH2 overexpression, and its effects were independent of proliferation and apoptosis. Surprisingly, the autophagosome formation was enhanced by EZH2 inhibition or knockdown in VSMCs, but reduced by EZH2 overexpression. On the other hand, the regulatory proteins for autophagosome formation, ATG5 and ATG7, were significantly increased in EZH2 inhibited or deficient VSMCs, and knockdown of ATG5 or ATG7 could largely restore VSMC growth and abolish autophagosome formation induced by EZH2 inhibited or knockdown. In addition, we identified that MEK–ERK1/2 signaling was also responsible for EZH2 in the regulation of ACD of VSMCs. Furthermore, when compared with normal counterparts, EZH2 expression levels were significantly decreased in the aortic wall of AD patients, while LC3II, the hallmark of autophagy, was enhanced. Therefore, our aforementioned results indicate that EZH2 is indispensable for VSMC homeostasis, which is beneficial in maintaining vascular integrity in order to reduce the occurrence of AD.

## Results

### Autophagy activation was accompanied by EZH2 expression level alteration in aortic wall of AD patients

**Fig. 1:**
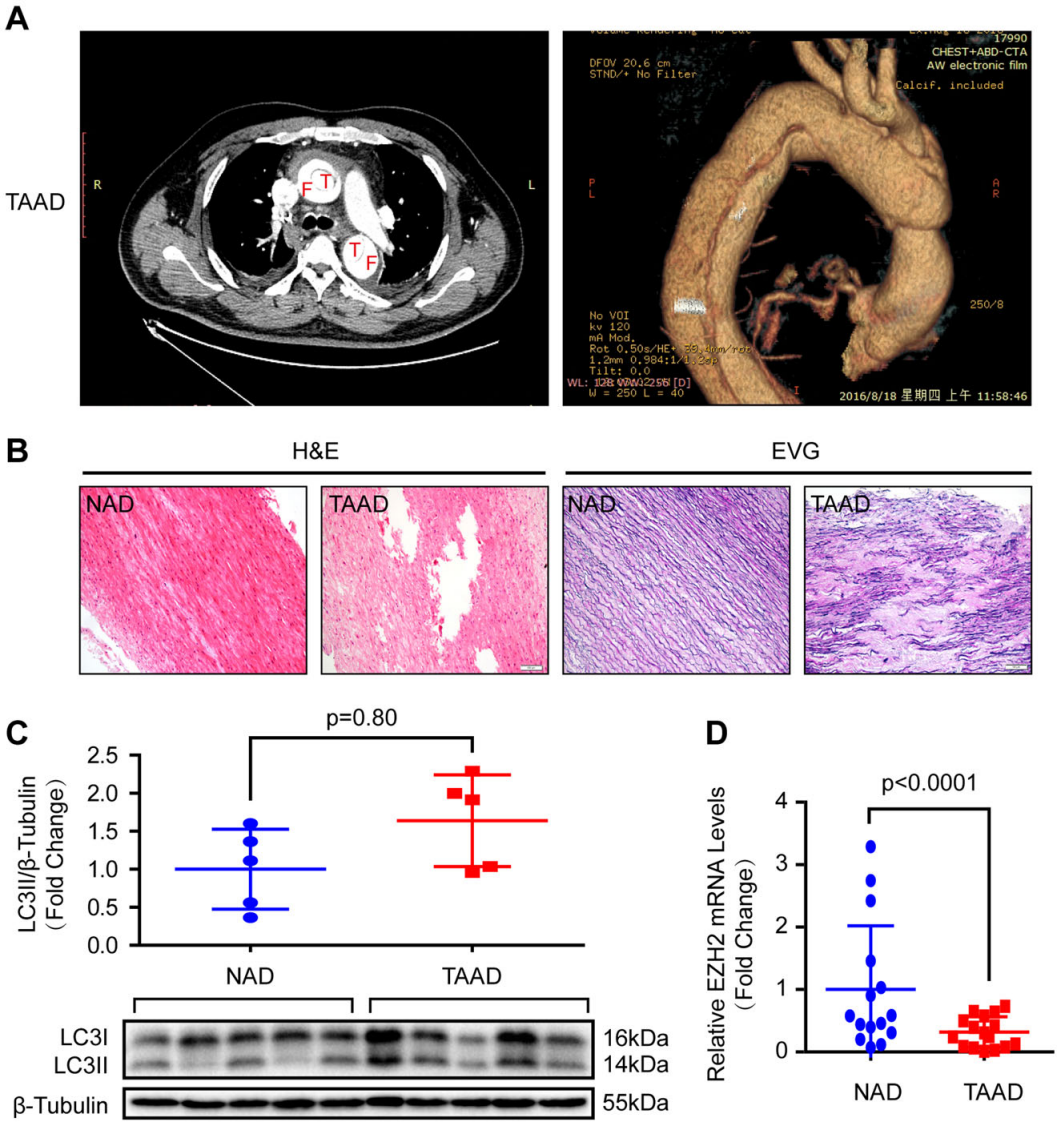
EZH2 expression level and autophagy were changed in the aorta of TAAD patients. **a** The representative iterative reconstruction CTA images of TAAD patients, T: true lumen; F: false lumen (*n* = 16). **b** The H&E and EVG staining of non-aortic dissection (NAD) and dissected human aorta (scale bar, 100 μm, *n* = 15–16). **c** The representative western blots and statistical results of LC3II/I in the aorta of NAD and TAAD patients, and β-tubulin serves as a loading control (*n* = 15–16). **d** The mRNA level of EZH2 was detected by using RT-PCR (*n* = 15–16)

**Fig. 2:**
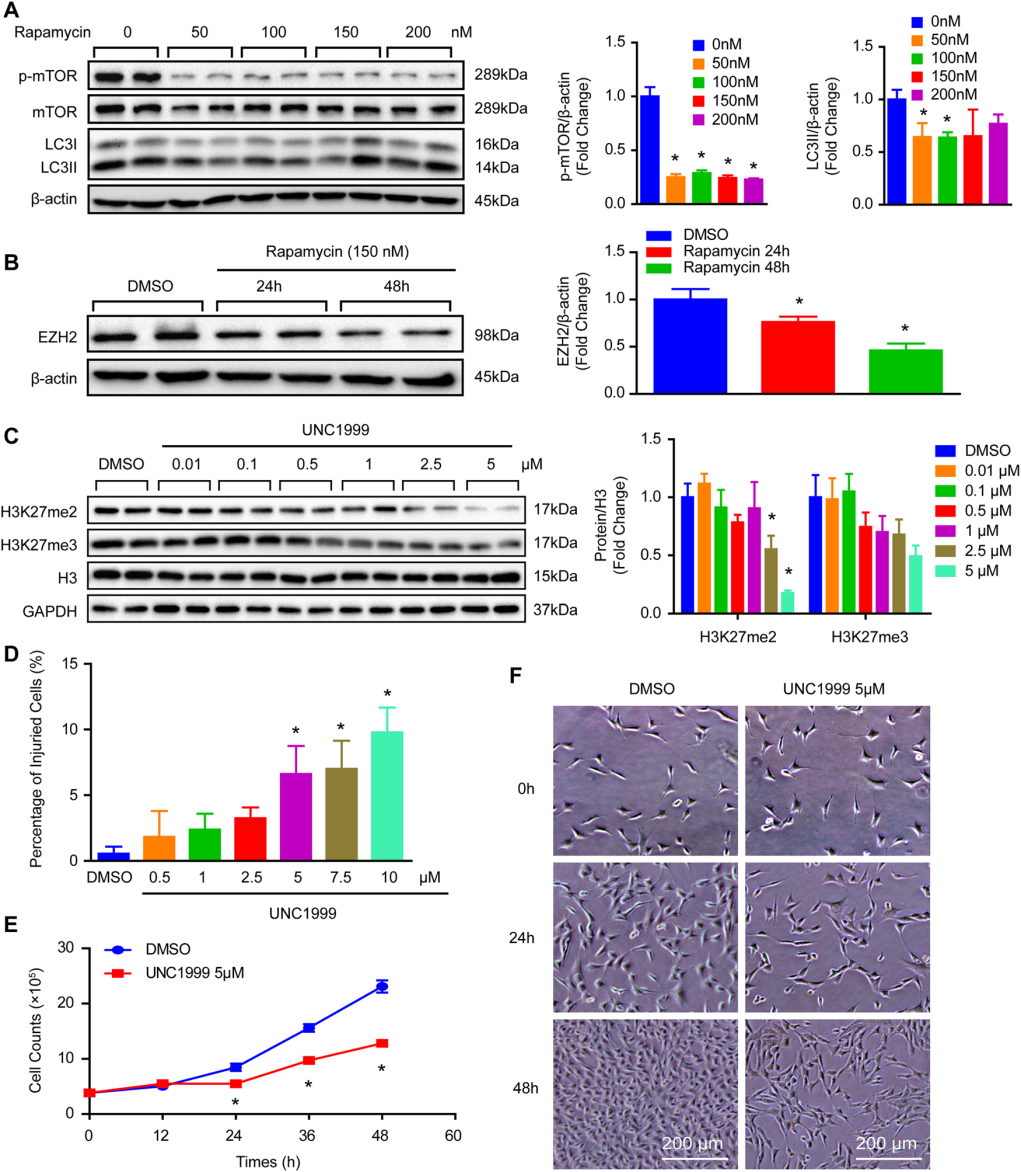
EZH2 inhibition by UNC1999 results in VSMC loss. **a** The protein level of phosphorylated mTOR and LC3II/I was detected by western blot in VSMCs after different concentrations of rapamycin (an autophagy inducer) treatment for 24 h (*n* = 4). *p < 0.05 vs. 0 nM of rapamycin. **b** The protein level of EZH2 was evaluated by western blot in VSMCs treated by 150 nM of rapamycin for indicated times (*n* = 4). β-Actin serves as a loading control. **c** Representative western blots and quantitative results of H3K27me2 and H3K27me3 in VSMCs treated by different UNC1999 concentration for 24 h (*n* = 4). GAPDH serves as a loading control. **d** The percentage of injured cells was evaluated by LDH kit after UNC1999 treatment for 48 h (*n* = 6). **e** The growth curve of VSMCs treated with DMSO or 5 μM of UNC1999 for indicated times (*n* = 3). **f** Representative images of VSMCs treated with UNC1999 or DMSO at different time point. *p < 0.05 vs. DMSO in **b–e**

Given that one of the typical morphological features of the aortic wall in patients with AD is VSMC loss and is closely related to VSMC autophagy^2,6^. We investigated the role of histone methyltransferase EZH2 in AD by collecting the aortic wall specimens of TAAD individuals and patients who underwent heart transplantation. The detailed clinical information of these patients are presented in Table S1. The results of computed tomography angiography (CTA) showed that the aortic diameter of TAAD patients was significantly increased, in addition, a true lumen and a false lumen were observed at the ascending and/or descending aorta (Fig. 1a). Hematoxylin–eosin (H&E) and Elastica van Gieson (EVG) staining were used to indicate that medial degeneration, as evidenced by fragmentation and loss of elastic fibers and VSMC loss is present in the aortic wall of TAAD patients (Fig. 1b). Recently, several studies have demonstrated that autophagy contributed to VSMC loss during AD occurrence^5,6^. In our TAAD samples, the protein levels of LC3II was enhanced (Fig. 1c). Next, to explore whether EZH2 is involved in these biological processes, we detected its expression level in the samples of normal and TAAD aortic wall. Our results showed that the mRNA level of EZH2 was dramatically decreased in TAAD samples (Fig. 1d). Moreover, we established an autophagy cell model by using a well-known autophagy inducer, rapamycin. Our results showed that in cultured MOVAS cells (mouse aorta SMCs, hereinafter referred to as VSMCs), phosphorylated mammalian targets of rapamycin (p-mTOR) were well inhibited by different concentrations of rapamycin (50–200 nM) for 24 h, and had decreased LC3II level, indicate autophagic flux going smoothly (Fig. 2a). In the present study, as more LC3 puncta was induced by 150 nM than 50 nM and 100 nM of rapamycin (data not shown), 150 nM of rapamycin was used to induce autophagy for the follow-up tests. Notably, EZH2 protein levels were remarkably decreased in VSMCs after 150 nM of rapamycin treatment for the indicated times, which implied that EZH2 may be involved in VSMC autophagy (Fig. 2b).

### EZH2 facilitates growth of VSMCs

**Fig. 3:**
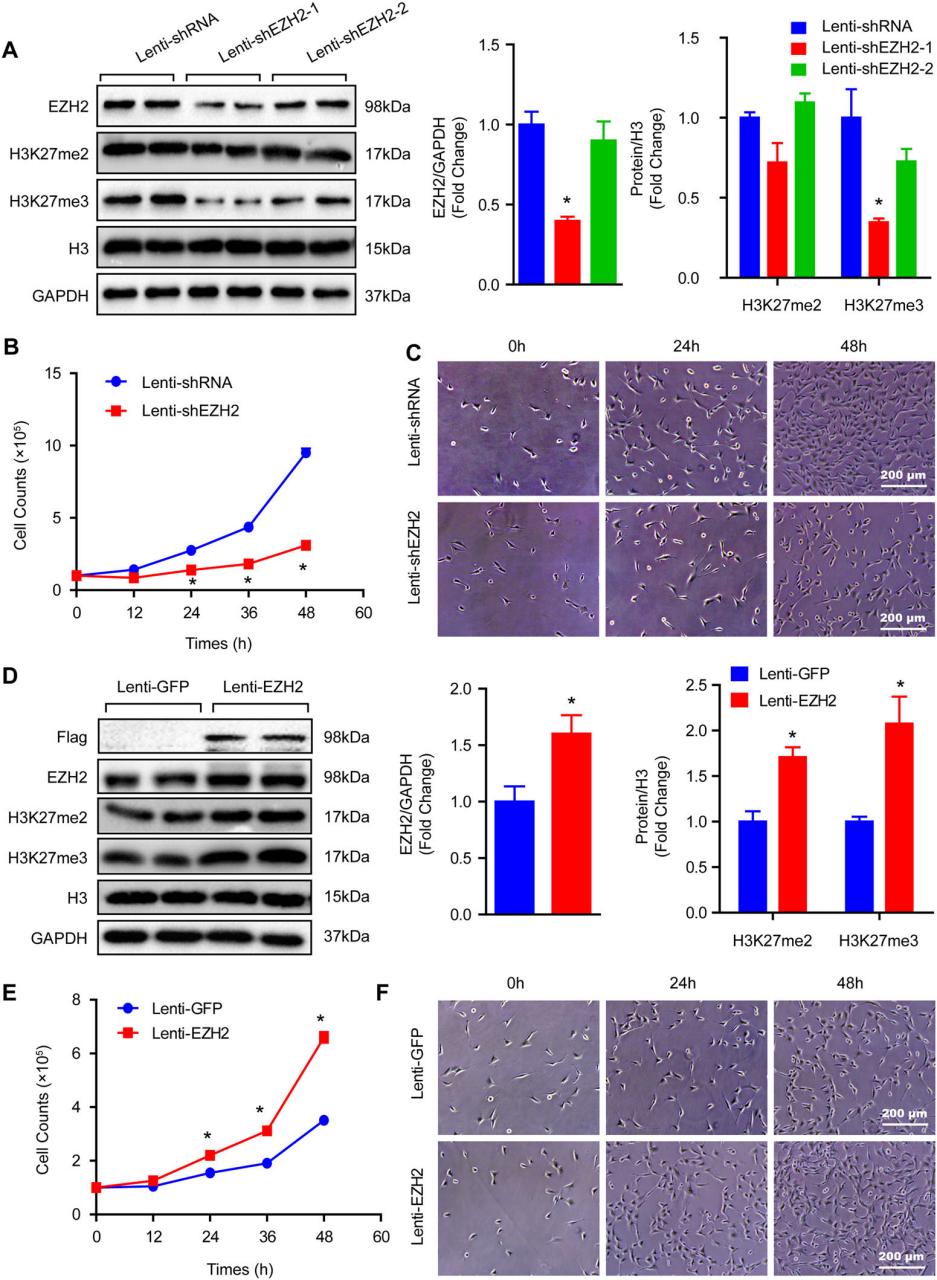
EZH2 positively regulates VSMC growth. **a** Protein level of EZH2, H3K27me2, and H3K27me3 were detected by western blot in VSMCs infected with lenti-shRNA or lenti-shEZH2. Left panel, representative western blots; right panel, quantitative results. GAPDH serves as a loading control (*n* = 4). **b** Growth curve of VSMC-infected lenti-shRNA or lenti-shEZH2 (*n* = 3). **c** Representative images of VSMCs under light microscopy. *p < 0.05 vs. lenti-shRNA in **a** and **b**. **d** The western blots of Flag, EZH2, H3K27me2, and H3K27me3 in the samples of VSMCs overexpressed GFP or EZH2 (left panel), and quantitative results of these blots showed at the right panel (*n* = 4). **e** The VSMC number was counted at indicated time point after lenti-GFP or lenti-EZH2 infected (*n* = 3). **f** Representative images of VSMCs under light microscopy. *p < 0.05 vs. lenti-GFP in **d** and **e**

To explore the effects of EZH2 on VSMCs, the EZH2 inhibitor, UNC1999, was used. Different concentrations of UNC1999 were used to stimulate VSMCs in order to assess the best dosage. Our results demonstrated that 5 μM of UNC1999 was the adequate concentration, as indicated by the robustly inhibited H3K27me2/3 levels and minor cell injuries detected by lactate dehydrogenase (LDH) release assay (Fig. 2c, d). Important to note that UNC1999 stimuli resulted in VSMC numbers visibly reducing as early as 24 h (Fig. 2e, f). To further address this concern, short hairpin RNA (shRNA) was used to knockdown EZH2 via lentivirus in VSMCs. The EZH2 expression level and methylation of H3K27 were verified by western blots, and the results showed that EZH2 protein levels and H3K27me3 were significantly reduced after shEZH2-1 lentivirus infection, while the knockdown efficiency of shEZH2-2 was less than satisfactory (Fig. 3a). Therefore, we used the lenti-shEZH2-1 (hereinafter referred to as lenti-shEZH2) for all follow-up experiments. Unexpectedly, the VSMC number was noticeably reduced after lenti-shEZH2 infection for the indicated time, as evidenced by the growth curve and cell images (Fig. 3b, c). Next, we were curious about whether EZH2 overexpression has any effects on VSMC growth. As presented in Fig. 3d, EZH2 was prominently increased in VSMCs infected with lenti-EZH2. When compared with the lenti-GFP group, H3K27me2 and H3K27me3 levels were also significantly increased after EZH2 overexpression (Fig. 3d). Notably, the results of the cell count at indicated time points showed that more cell numbers were observed in lenti-EZH2 than in the lenti-GFP group as early as 24 h (Fig. 3e, f).

### Cell proliferation and apoptosis are not involved in the role of EZH2 on VSMCs

As we known, cell proliferation and apoptosis are the primary factors affecting the growth of cells. Thus, we first evaluated whether VSMC proliferation was affected by EZH2. The proliferation markers PCNA and phosphorylated histone H3 (p-H3) were detected by western blot, and Ki67 was measured via immunofluorescence analysis. Our results demonstrated that EZH2 inhibition by UNC1999 had no effect on PCNA, p-H3, and Ki67 protein levels (Figure S1A and S1B). Flow cytometry was applied to check the cell cycle of VSMCs. In that process we found that the cell percentage in neither G0/G1, S, nor G2/M phase had an obvious difference between the dimethyl sulfoxide (DMSO) and UNC1999 group (Figure S1C and S1D). These results suggest that EZH2 had no effects on VSMC proliferation. In addition, VSMC apoptosis was analyzed via flow cytometry. Its results also showed that there was no obvious difference in the early and late apoptosis periods between DMSO and UNC1999 stimulation (Figure S1E). Similarly, EZH2 knockdown or overexpression had no effect on VSMC proliferation and apoptosis (Figures S2 and S3).

### EZH2 suppresses autophagosome formation in VSMCs

**Fig. 4:**
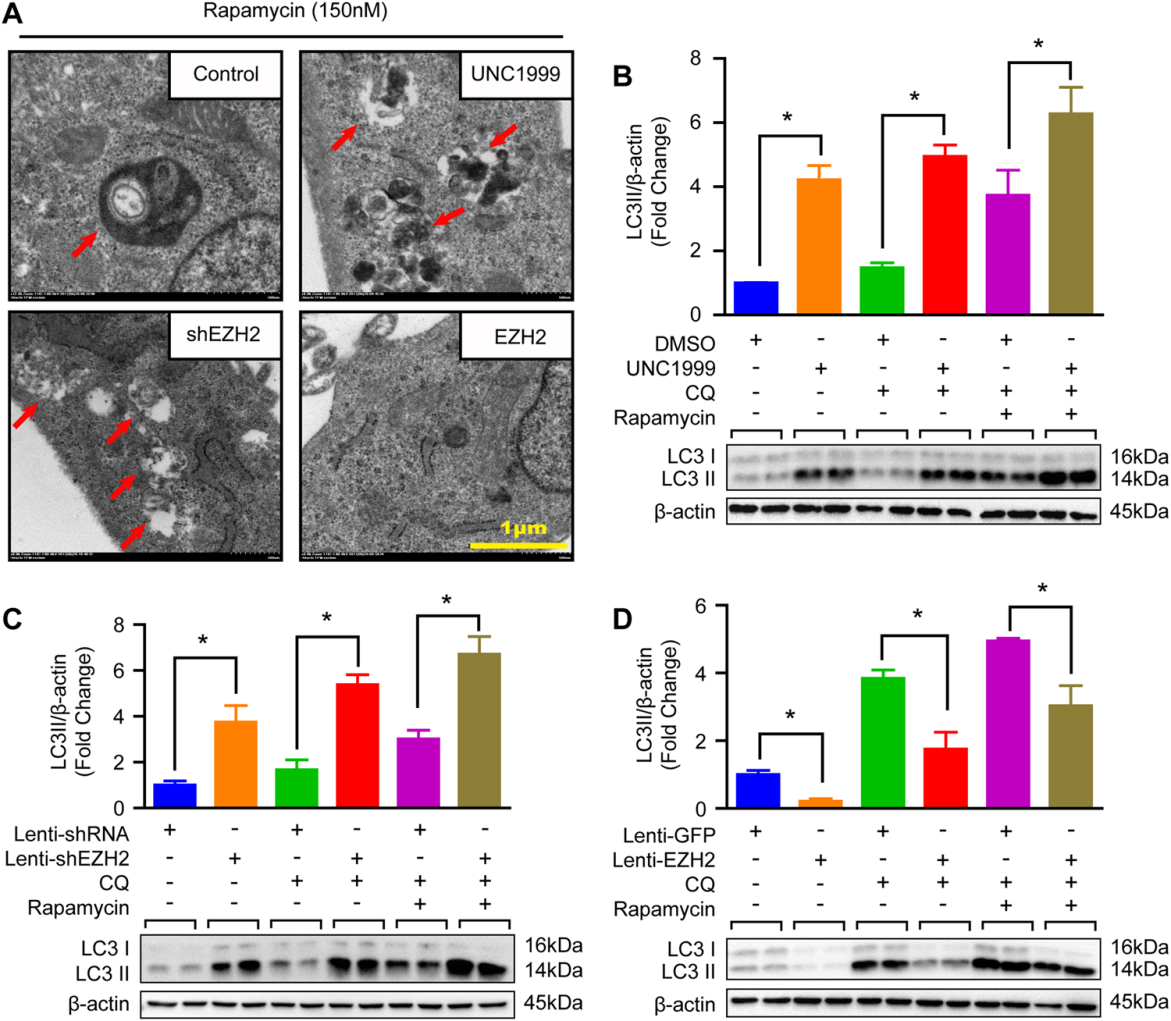
EZH2 inhibited autophagosome formation in VSMCs. **a** The representative images of autophagic vacuole under a transmission electron microscope and red arrow indicates autophagic vacuole (scale bar, 1 μm). **b–d** The protein levels of LC3II/I were detected by using western blot in the indicated groups (*n* = 4), and the quantitative results were shown at the upper panel respectively. β-Actin serves as a loading control. *p < 0.05

Except for proliferation and apoptosis, autophagy is another well-known biological process associated with cell growth. As we have mentioned in Fig. 2b, reduced EZH2 expression levels during autophagy activation indicated that EZH2 may participate in this biological process. To test this hypothesis, we started by detecting autophagy vacuoles by using electron microscopy. As presented in Fig. 4a, double-membraned vacuoles containing fragments of cytoplasmic components were observed in control and EZH2 knockdown or inhibition VSMCs, and more vacuoles were found in EZH2 knockdown or inhibited VSMCs. However, almost no vacuole was detected in EZH2 overexpression VSMCs (Fig. 4a). In addition, EZH2 knockdown or inhibition resulted in LC3II levels being remarkably elevated, while EZH2 overexpression decreased LC3II protein levels in VSMCs (Fig. 4b–d). Important to note, when compared with their controls, chloroquine (CQ, blocking degradation of autophagosomal components) alone or CQ with rapamycin treatment led to more LC3II accumulation in EZH2 inhibited or knockdown VSMCs, but an opposite scenery was detected in EZH2 overexpressed VSMCs, which indicated that EZH2 negatively regulated autophagosome formation (Fig. 4b–d). We next infected lenti-mCherry-EGFP-LC3 reporter plasmid into VSMCs to further address the effects of EZH2 on autophagy flux. Given that EGFP is acid sensitive, while mCherry is acid insensitive. Thus, both EGFP and mCherry existed in autophagosomes, but only mCherry was included in autolysosomes. As shown in Figure S4A, neither in EZH2 inhibited/knockdown nor in EZH2 overexpressed VSMCs, the ratio of autophagosomes (indicated by both EGFP and mCherry positive) number to autolysosomes (indicated by only mCherry positive) number has significant difference compared with their counterparts, respectively. These results indicated that EZH2 inhibits autophagosome formation rather than affect autophagy flux.

### EZH2 controls autophagosome formation by regulating ATG5 and ATG7 expression to manage ACD of VSMCs

**Fig. 5:**
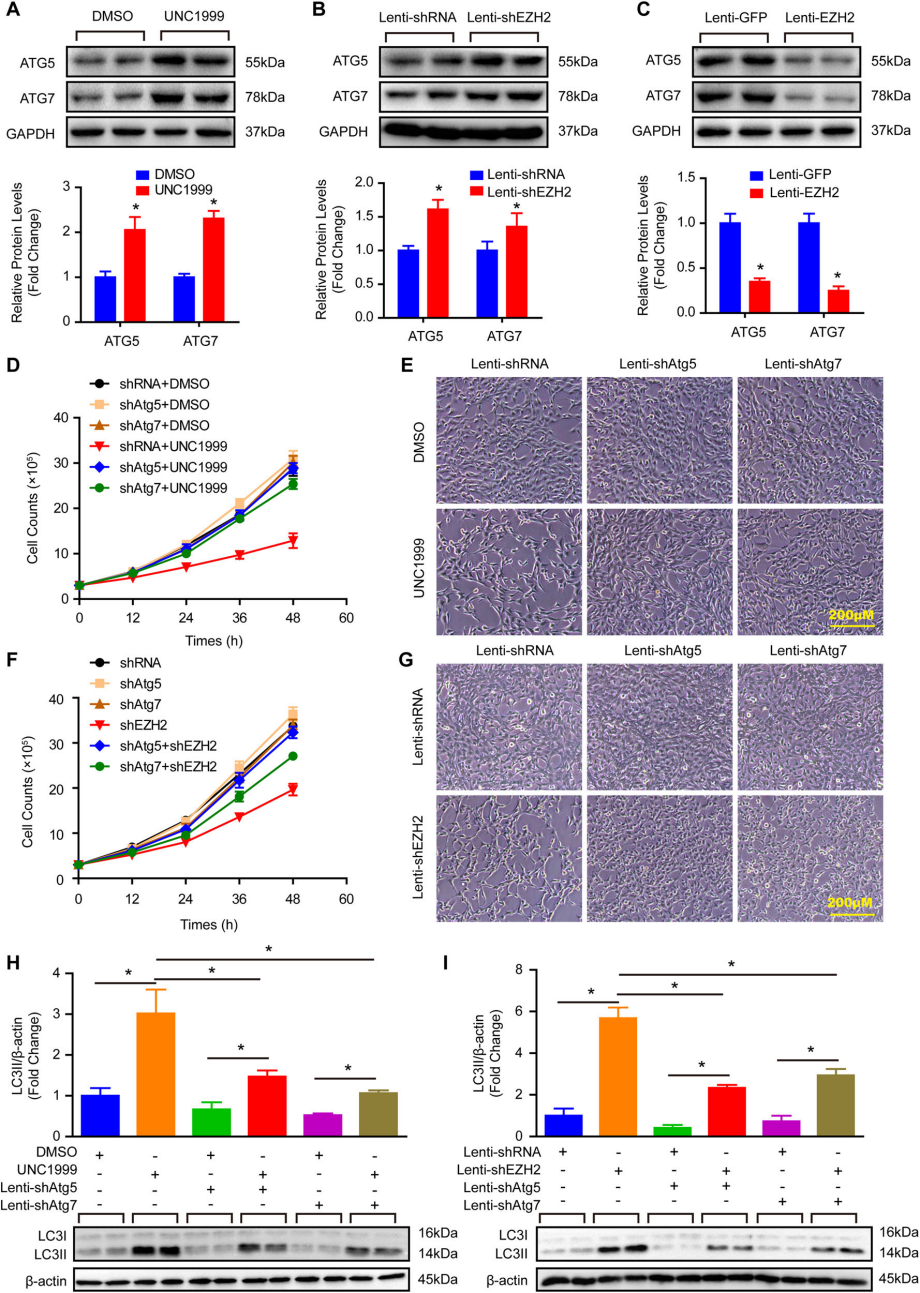
Inhibition autophagy by knockdown of ATG5 or ATG7 largely reverses EZH2 deficiency or inhibition induced ACD of VSMCs. **a–c** The protein levels of ATG5 and ATG7 were verified by western blot in VSMCs treated with indicated stimulus for 24 h; upper panel, representative blots; lower panel, quantitative results of blots (*n *= 4). GAPDH serves as a loading control. *p < 0.05 vs. DMSO (**a**) or lenti-shRNA (**b**), or lenti-GFP (**c**). **d**,** f** The VSMC cell number was counted in the indicated time point after treated with indicated stimulus (*n* = 3). **e**,** g** The representative images of VSMCs under light microscopy after indicated stimulus treated for 48 h (scale bar, 200 μm). **h, i** The LC3II/I protein level was evaluated by western blot; lower panel, representative blots; upper panel, quantitative results of blots (*n* = 4). β-Actin serves as a loading control. *p < 0.05

The aforementioned results indicate EZH2 induced excessive autophagosome formation that resulted in the loss of VSMCs. Previous studies have revealed that excessive autophagy could induce cell death which is defined as ACD^10^. Is it ACD induced by EZH2 causing VSMC loss? To further address this concern, we firstly detected the protein levels of autophagosome formation regulatory molecules, ATG5 and ATG7, in EZH2 inhibited, knockdown, and overexpression groups. Our results revealed that both ATG5 and ATG7 were increased during EZH2 inhibition and knockdown (Fig. 5a, b). Nevertheless, both of them were decreased strikingly in the EZH2 overexpression group in comparison with the control (Fig. 5c). On the other hand, we knockdown ATG5 or ATG7 in VSMCs with or without EZH2 deficiency, respectively. The knockdown efficiency was verified by western blots, and the results showed that both ATG5 and ATG7 were significantly reduced after shATG5-1 and shATG7 lentivirus infection (Figure S4B and S4C), while the knockdown efficiency of shATG5-2 was not satisfied (Figure S4B). Therefore, we used the lenti-shATG5-1 (hereinafter referred to as lenti-shATG5) for all follow-up experiments. Our results showed that ATG5 or ATG7 knockdown largely rescued VSMCs from cell death induced by EZH2 inhibited or knockdown (Fig. 5d–g). Moreover, the increased protein levels of LC3II were also offset by ATG5 or ATG7 knockdown in VSMCs with EZH2 inhibition or deficiency, which indicated autophagy weakening within in these VSMCs (Fig. 5h, i). Thus, our results indicate that EZH2 controls autophagosome formation by regulating ATG5 and ATG7 expression to manage ACD of VSMCs.

### The effects of EZH2 on VSMC autophagy were independent of AMPK, mTOR, and AKT signaling pathway

**Fig. 6:**
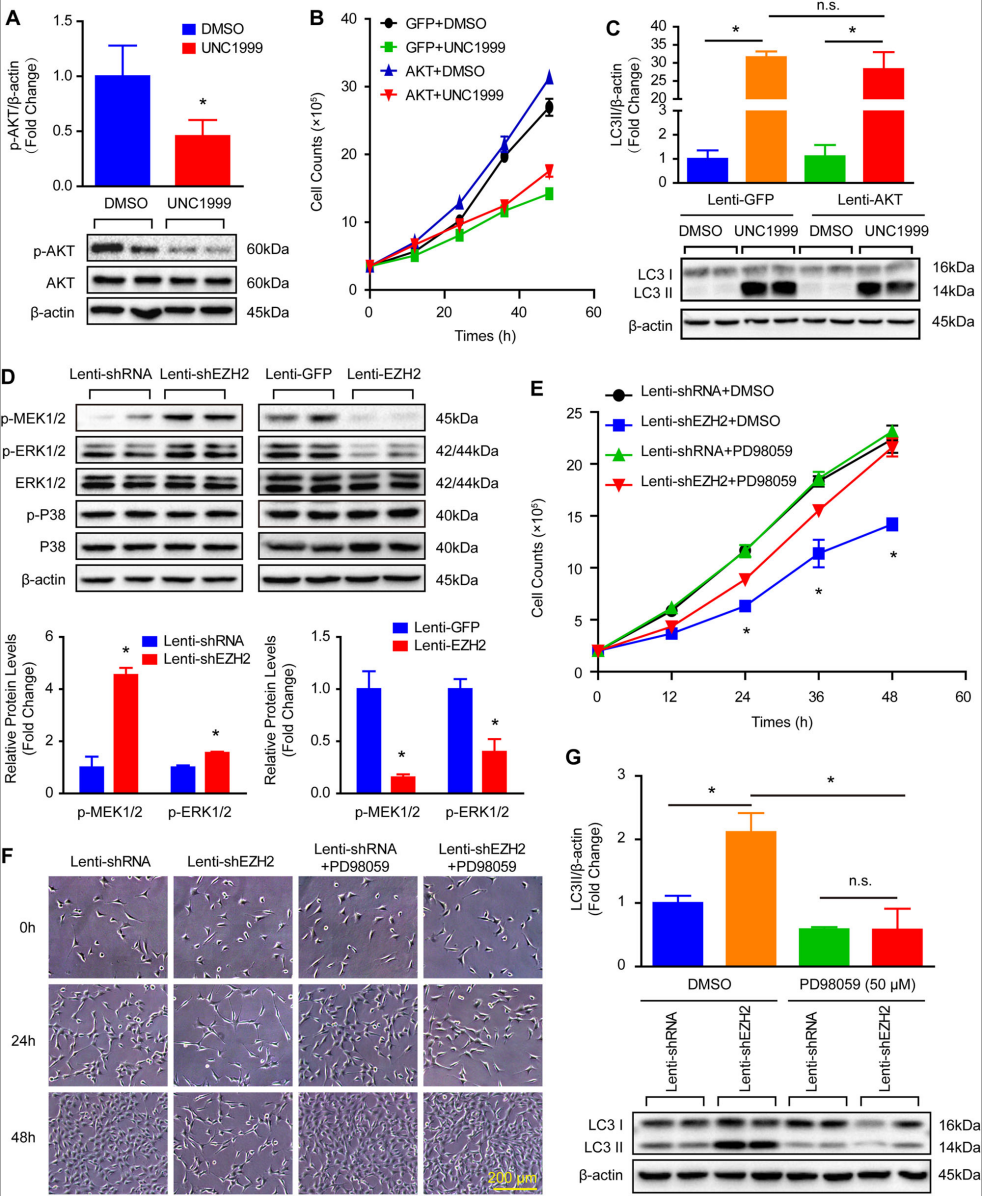
The role of EZH2 in ACD of VSMCs is largely dependent on the MEK–ERK1/2 signaling pathway. **a** The phosphorylation levels of AKT in VSMCs treated with EZH2 inhibitor UNC1999 for 24 h (*n* = 4), *p < 0.05 vs. DMSO. **b** The growth curve of AKT overexpressed VSMCs treated with or without UNC1999 (*n* = 3). **c** The protein levels of LC3II/I in the AKT overexpressed VSMCs treated with or without UNC1999 for 24 h was evaluated by western blot (*n* = 4). **d** The phosphorylation levels of MEK1/2, ERK1/2, P38 in VSMCs with EZH2 knockdown or overexpression (*n* = 4), *p < 0.05 vs. lenti-shRNA or lenti-GFP. **e** The growth curve of VSMCs treated with indicated stimulus (*n* = 3, *p < 0.05 vs. lenti-shEZH2 + PD98059). **f** The representative VSMC images under light microscopy after treated with indicated stimulus (scale bar, 200 μm). **g** The protein levels of LC3II/I was detected by using western blot in VSMCs treated with indicated stimulus for 24 h (*n* = 4). β-Actin serves as a loading control. *p < 0.05, n.s. indicated no significance

Except function as histone methyltransferase, EZH2 was also involved in several signaling pathway modulation^18–21^. Thus, we are curious about whether EZH2 modulate canonical autophagy signaling pathways to affect VSMCs. For this we investigated the adenosine 5′-monophosphate-activated protein kinase (AMPK) signaling and mTOR signaling pathway, both well known to be involved in autophagy^19^. Our results showed that phosphorylation levels of AMPKα, mTOR, and S6 displayed no obvious changes after EZH2 knockdown, inhibition, or overexpression (Figure S5A–S5F). These data indicated that anti-autophagy effects of EZH2 might not be associated with the AMPKα, mTOR, and S6. On the contrary, we found that the phosphorylation levels of AKT were decreased by EZH2 inhibition in VSMCs (Fig. 6a). Thus, to determine whether AKT mediated the anti-autophagy role of EZH2 in VSMCs, we treated AKT overexpressed VSMCs with EZH2 inhibitor UNC1999, which in turn significantly increased AKT and decreased H3K27me2 and H3K27me3 were observed (Figure S6A and S6B). More importantly, our results showed that AKT overexpression accelerated VSMC growth, but the detrimental effect of EZH2 inhibition on VSMCs was not rescued by AKT overexpression, as evidenced by no differences in the cell numbers between AKT with UNC1999 and only UNC1999-treated groups (Fig. 6b and Figure S6C). Furthermore, AKT overexpression also has no impact on UNC1999-induced autophagy, as indicated by the comparable LC3II protein level in VSMCs treated with UNC1999 or UNC1999 with AKT overexpression (Fig. 6c). These results indicate that AKT is suppressed by EZH2 inhibition, but does not mediate the anti-autophagy effects of EZH2 on VSMCs.

### MEK–ERK1/2 signaling pathway mediated the effects of EZH2 on ACD of VSMCs

Given that MEK–ERK1/2 signaling pathway is also one of the most important signals when it comes to regulation autophagy^20^, we verified whether MEK–ERK1/2 signaling was involved in the process of EZH2 on VSMC autophagy. Our results showed that the phosphorylation levels of MEK1/2 and ERK1/2, but not P38, were dramatically increased in EZH2 knockdown VSMCs, when compared with the non-target shRNA group (Fig. 6d). Conversely, overexpression of EZH2 obviously reduced the levels of MEK1/2 and ERK1/2 phosphorylation when compared with those of GFP control (Fig. 6d). The above findings suggested that MEK1/2 or ERK1/2 inhibition might neutralize the negative effects of EZH2 knockdown or inhibition on VSMC growth and autophagy. To test this hypothesis, we firstly stimulated VSMCs with different concentrations of PD98059 (an MEK1 inhibitor) to get the optimum concentration of this drug. As showed in Figure S7A, 50 μM or higher concentrations of PD98059 has satisfactory inhibition effects on MEK1 activity, as evidenced by almost nonexistent phosphorylation levels of ERK1/2. Thus, to lower cytotoxicity, 50 μM of PD98059 was used for all following experiments. PD98059 almost totally offset the increased level of phosphorylated ERK1/2 induced by EZH2 knockdown and inhibition (Figure S7B). Remarkably, EZH2 deficiency or inhibition-mediated VSMC loss was largely reversed by PD98059, which was evidenced by more VSMC counts (Fig. 6e, f, Figure S7C and S7D). In addition, the elevated LC3II protein level in VSMCs with EZH2 knockdown or inhibition was also greatly abolished by PD98059 (Fig. 6g and Figure S7E). Thus, our findings suggested that EZH2 negatively modulated VSMC autophagy and then facilitated its growth, at least in part by inhibiting MEK–ERK1/2 signaling.

**Fig. 7:**
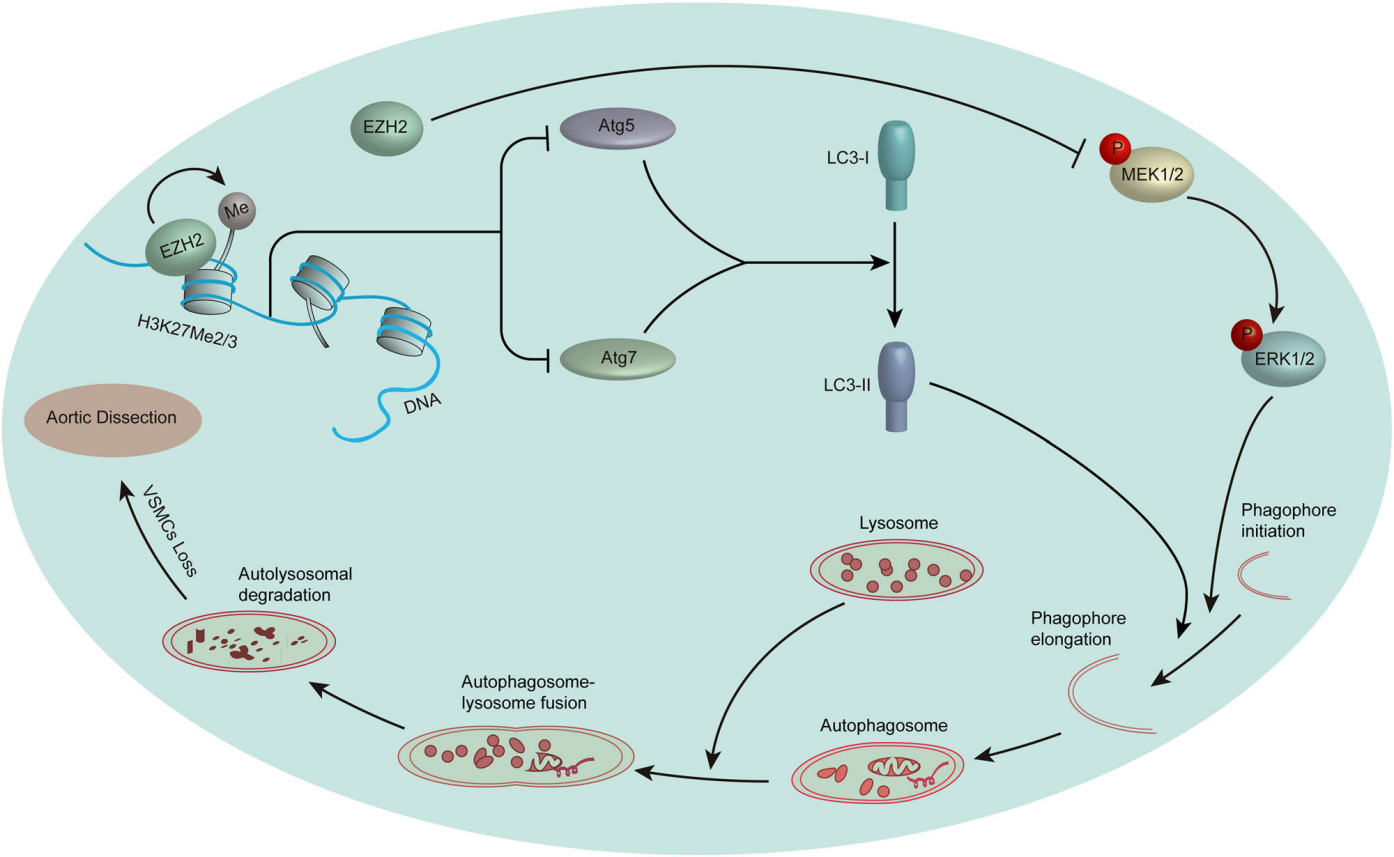
Proposed mechanism of EZH2 regulates ACD of VSMCs and AD occurrence. In the aorta of AD patients, reduced EZH2 expression results in H3K27me2 and H3K27me3 level decrease, which leads to increase expression of ATG5 and ATG7. On the other hand, the MEK–ERK1/2 signaling pathway is activated by decreased EZH2. Enhanced ATG5 and ATG7 expression and MEK–ERK1/2 signaling activation jointly induce excessive autophagosome formation and then result in ACD of VSMCs, which ultimately accelerates AD occurrence

Taken together, all the aforementioned results indicate that EZH2 may play a critical role during VSMC loss or even AD occurrence via regulating autophagy, which is largely mediated by regulating ATG5 and ATG7 expression and the MEK–ERK1/2 signaling pathway (Fig. 7).

## Discussion

An increasing number of studies have demonstrated that autophagy of VSMCs is profoundly involved in the development of AD. Accordingly, understanding of such mechanisms like those responsible for VSMC autophagy will provide new strategies in the prevention and treatment of AD. In this study, we found that EZH2 contributed to VSMC loss, which was independent of cell proliferation and apoptosis. Further studies revealed that inhibited autophagy by ATG5 or ATG7 knockdown largely reversed EZH2 inhibition or deficiency induced ACD of VSMCs. Furthermore, we uncovered that MEK–ERK1/2, but not AMPKα, mTOR, or AKT signaling pathway, mediated the function of EZH2 on ACD of VSMCs. More importantly, we detected reduced EZH2 expression levels in the aorta of patients with AD, when compared with their normal counterparts. Based on these results, we surmise that decreased EZH2 expression in the aorta of AD patients may partially responsible for VSMC loss in the aorta, and activation EZH2 could be a promising therapeutic target for AD.

An AD caused by an intimal tear or rupture of the vasa vasorum is a life-threatening condition^1,22^. The main feature of AD is medial degeneration, as evidenced by fragmentation of elastic fibers and VSMC loss^22^. Historically, cell death has been identified based on morphology. According to that classification they are Type I (apoptosis), Type II (autophagic cell death), and Type III (necrosis)^10,23^. VSMC apoptosis accounts as the primary reason of this loss^22^. Recently, Wang et al.^6^ demonstrated that more autophagic vacuoles and increased expression of autophagic markers were observed in the aortic wall of AD patients^6^, which is also validated by our data that are shown in Fig. 1. Even more, their results also showed that blockage of autophagy could suppress PDGF-induced phenotypic switch of SMCs and inhibit starvation-induced apoptosis of SMCs^6^. In addition, conditional deletion of myocardin from the SMCs of mice triggered endoplasmic reticulum (ER) stress and autophagy in SMCs, leading these mice to develop arterial aneurysms, dissection, and rupture^5^. Overexpression of MYH11 was able to increase ER stress and autophagy, and mutations in MYH11 caused syndrome-associated thoracic aortic aneurysm/ADs^2,24^. These results indicate that autophagy is crucial for VSMC function and AD occurrence. Thus, it is critical to clarify the mechanisms that regulate VSMC autophagy. In this study, we found that EZH2 expression levels decreased in the aortic wall of AD patients, and reduced EZH2 protein levels were detected in VSMCs treated with rapamycin, an autophagy inducer. More importantly, we identified that autophagic activity was enhanced by EZH2 knockdown or inhibition, while attenuated by EZH2 overexpression in VSMCs. These results suggest that EZH2 is a novel autophagy regulator in VSMCs.

Autophagy is a dynamic and highly regulated process that is essential for maintaining cellular homeostasis^25^. Previous studies have revealed that autophagy is a double-edge sword in multiple diseases including tumorigenesis, neurodegeneration, and ischemic injury^26–29^. A series of studies reported that strategies that enhance autophagy can promote survival in response to milder stress, such as brief hypoxia and low levels of oxidative stress, whereas severe stress^30,31^, such as prolonged hypoxia or subsequent reperfusion, result in excessive autophagy^18,32^, which may cause cell death by triggering excessive self-digestion of essential proteins and organelles. Yutaka Matsui et al.^30^ demonstrated that autophagy enhanced in the heart of mice after short-term ischemia reperfusion and cardiac injury was attenuated in autophagy deficient mice^30^. Severe hypoxia induce progressive autophagy activation, and knockdown of Beclin1 could suppress autophagy and ameliorate cell survival^32^. These results implied that extensive activation of autophagy could induce ACD resulting in disease deterioration. According to the recommendation of the nomenclature committee on Cell Death 2012, the term ACD is defined to indicate cell death instance that is mediated by autophagy and can be suppressed by the inhibition of the autophagic pathway by chemicals and/or genetic means^10^. In this research, we found that both ATG5 and ATG7 levels are significantly increased in EZH2 inhibited/knockdown VSMCs, while their levels are decreased when EZH2 is overexpressed. More importantly, knockdown of ATG5 and ATG7 could largely reverse VSMC loss and abolish autophagosome formation induced by EZH2 inhibition or knockdown. Thus, our data demonstrated that EZH2 controlled autophagosome formation by regulating ATG5 and ATG7 expression to manage ACD of VSMCs and contribute to prevent AD occurrence.

Previous studies have revealed the crucial and extensive role of ACD in tumorigenesis, neurodegeneration, and ischemic injury^26–29^. In addition, ACD is also involved in progression of cardiovascular disease. Knaapen et al.^33^ examined the cardiac tissue of patients in the terminal stage of heart failure as a consequence of ischemic cardiomyopathy or dilated cardiomyopathy and discovered that cardiomyocytes in heart failure are caspase-independent ACD rather than apoptotic cell death. Stefan Hein et al.^34^ discovered that ACD induced cell loss contributes significantly to the progression of left ventricular (LV) systolic dysfunction in patients with valvular aortic stenosis and differing degrees of LV systolic dysfunction. In the present study, we discovered that ACD contributes to VSMC loss and plays a potential role in AD occurrence. More importantly, we illustrate that EZH2 functions as a crucial regulatory molecule of ACD in VSMCs, which could be a potentially therapeutic target in AD treatment. Furthermore, our study also offer a more comprehensive understanding of the role of ACD in disease.

EZH2 is not only known as a histone methyltransferase conducting H3K27me1/2/3 and gene transcriptional repression but is also involved in several signaling pathway modulation^21,35,36^. In breast cancer cells and non-small cell lung carcinoma cells, MEK–ERK1/2 signaling pathway activation leads to EZH2 overexpression^21^, while another study demonstrated that downregulation of EZH2 is associated with activation of the Src–Raf–ERK signaling pathway^35^. Our results demonstrated that EZH2 negatively regulates the MEK–ERK1/2 pathway, but not mTOR, AMPKα, or AKT pathways in the management of autophagy and VSMC loss. Previous studies had a well-defined relationship between ERK1/2 and autophagy^35,36^. It has been reported that inhibition of ERK1/2 phosphorylation by using the specific MEK inhibitor could partially abrogate the autophagy^37^, which is identified by our data in the present study. Similarly, Xiao et al.^38^ demonstrated that ERK1/2 knockdown could decrease autophagy and autophagy-related protein 7 (ATG7) expression to promote liver steatosis. On the other hand, ERK2 was able to interact with ATG proteins via its substrate-binding domains to localize to the extra-luminal face of autophagosomes, which resulted in enhanced ERK phosphorylation to further accelerate autophagy^36^. In our study, we found that the MEK–ERK1/2 signaling pathway and ATG5 and ATG7 were regulated by EZH2 at the same time, and we also did not rule out the possibility of MEK–ERK1/2 signaling regulating ATG5 and ATG7 expression or their interaction in VSMCs. On the other hand, recent studies have shown that the MEK–ERK1/2 signaling pathway is also a contributor to other pathophysiological processes of AD, including VSMC phenotype switch^39^, apoptosis^40^, and proliferation inhibition^41^. Although we have demonstrated that EZH2 has no effects on VSMC apoptosis and proliferation, whether EZH2 could regulate VSMC phenotype switch via MEK–ERK1/2 signaling remains unknown. In addition, Wang et al.^42^ found that phospho-ERK1/2 was increased significantly in the aortic wall from patients with AD^42^. Moreover, their results showed that the MEK–ERK1/2 pathway was involved in Ang-II-induced phenotypic differentiation and matrix metalloproteinase-2 mRNA expression in aortic adventitial fibroblasts, which could lead to disorder the delicate balance of ECM metabolism in the aortic wall^42^.

In closing, our data demonstrate that EZH2 affects VSMC loss and even pathologic process of AD via regulating ACD of VSMCs, which is mediated by suppressing ATG5 and ATG7 expression and MEK–ERK1/2 signaling. This study may broaden our understanding of the molecular mechanisms underlying VSMC loss in AD patients. Furthermore, our results identified that EZH2 might be a novel therapeutic target for the prevention of AD.

## Methods and materials

### Human aorta samples

Thirty-one biopsies of aortic samples were collected from 16 patients with TAAD and 15 patients underwent heart transplantation (served as control). Patients’ clinical information are described in Table S1. Informed consent was obtained from all subjects. This study was approved by the Tongji Hospital, Tongji Medical College, Huazhong University of Science and Technology Review Board in Wuhan, China.

### Antibodies

The antibodies used in this study including EZH2 (#5246), H3K27me2 (#9728), H3K27me3 (#9733), p-MEK1/2 (#9154), ERK1/2 (#4695), p-ERK1/2 (#4370), P38 (#8690), p-P38 (#4511), mTOR (#2983), p-mTOR (#5536), S6 (#2317), p-S6 (#5364), p-AMPKα (#2535), AMPKα (#5831), LC3II/I (#12741), FLAG (#2368), ATG5 (#12994), ATG7 (#8558), GAPDH (#5174), and β-actin (#8457) were obtained from Cell Signaling Technology. Antibodies against Histone H3 (ab1791) and Ki67 (ab16667) were purchased from Abcam. PCNA (GTX100539) and p-Histone H3 (sc-8656-R) were got from Genetex and Santa Cruz, respectively.

### Plasmids

The EZH2 overexpression and knockdown plasmids were kindly presented by Dr. Ke Chen (Tongji Hospital, Tongji Medical College, HUST)^43^. Double-strand oligonucleotides of shRNA targeting to mouse ATG5 and mouse ATG7 were cloned into pLKO.1 plasmids, at *Age*I and *Eco*RI restriction enzyme sites, whose target sequences were: shATG5-1: AGCCGAAGCCTTTGCTCAATG; shATG5-2: GCAGAACCATACTATTTGCTT; shATG7: GTCCTTCCATGTGCACTAATC.

### Histological analysis

The human aorta sample was obtained after operation, and the samples were quickly put in 10% formalin for fixation after being washed with cold saline solution. After 3 days of fixation, the tissue specimens were dehydrated and embedded in paraffin by following standard histological procedures. Subsequently, sectioning was performed and slices were stained with H&E for morphological examination as previously described^44,45^. EVG staining was performed for elastic fiber assessment. The images were captured by using an Olympus light microscope BX53 system.

### Cell culture and treatments

The MOVAS cells (ATCC^®^ CRL-2797^TM^), a mouse VSMC cell line, were cultured with Dulbecco's modified Eagle's medium (DMEM)/high glucose (SH30022.01; Hyclone) supplemented with 10% foetal bovine serum (SH30084.03; Hyclone), and 1% penicillin–streptomycin (15140-122; ThermoFisher Scientific). Cells were infected with lenti-mCherry-GFP-LC3, lenti-shRNA, lenti-shEZH2, lenti-GFP, lenti-EZH2, lenti-AKT, lenti-shATG5, or lenti-shATG7 lentivirus, respectively. After lentivirus infection for 48 h, the cells were treated with puromycin (2 μg/ml, P8833; Sigma-Aldrich) to select cells infected by lentivirus. In addition, MOVAS cells were stimulated with rapamycin (150 nM, S1039; Selleck), Chloroquine (20 μM, C6628; Sigma-Aldrich), UNC1999 (5 μM, S7165; Selleck), or PD98059 (50 μM, #9900L; Cell Signaling Technology), respectively. The LDH release was detected by using a cytotoxicity LDH assay kit (CK12; Dojindo). As previous described, Cell counting methodologies and averaging were achieved by standardized protocols^46^. MOVAS cells were cultured in serum-free DMEM/high glucose for 12 h. After that, 2 × 10^5^ cells were planted into six-well plates in DMEM/high glucose media with 10% fetal bovine serum. At 12, 24, 36, 48 h after planted, cells were trypsinized and single-cell suspensions were made for cell counting, respectively. Cell counting was performed by a single observer unaware of sample identity. Intra-assay coefficients of variation were obtained by accessing reproducibility of cell counts from three replicate wells.

### Western blot

The total protein from aortic tissues or MOVAS cells was extracted by RIPA as previously described^44,45,47,48^. After the protein denaturation, 20 μg of total protein was loaded and separated by sodium dodecyl sulfate polyacrylamide gel electrophoresis. Then, the protein was transferred to a polyvinylidene fluoride membrane (Millipore, IPVH00010), which was then blocked by 5% non-fat milk. Subsequently, the membrane was incubated with indicated primary antibody overnight at 4 °C. After incubating with the peroxidase-conjugated secondary antibody (Jackson ImmunoResearch Laboratories, 111-035-003, at 1:25000 dilution), the protein signals were detected by using the ChemiDoc^TM^ XRS+ system (Bio-Rad).

### Real-time PCR

Real-time PCR was performed as previously reported^45,49–52^. Briefly, total mRNA was isolated by using TRI Reagent^®^ Solution (AM9738; ThermoFisher Scientific). Then, the mRNA was reversely transcribed into cDNA by using a transcriptor first strand cDNA synthesis kit (4896866001; Roche). The relative mRNA levels of EZH2 was detected by CFX connect™ real-time PCR detection system (Bio-Rad) using iQ™SYBR^®^ green supermix (1708884; Bio-Rad). Primers used in this study were EZH2 forward primer 5′-CGATGATGATGATGGAGACG-3′ and EZH2 reverse primer 5′-GCTGTGCCCTTATCTGGAAA-3′, GAPDH forward primer 5′-GAGTCAACGGATTTGGTCGT-3′ and GAPDH reverse primer 5′-TTGATTTTGGAGGGATCTCG-3′.

### Immunofluorescence analysis

The cultured MOVAS cells infected with lenti-shEZH2, lenti-shRNA, lenti-GFP, or lenti-EZH2, or treated with UNC1999 or DMSO were fixed with 4% paraformaldehyde for 15 min. 0.2% of Triton X-100 was used for penetrating the cell membrane. After that, the samples were blocked by using 8% of goat serum for 60 min at room temperature. Subsequently, the primary antibody Ki67 (ab16667, 1:250 dilution) was incubated for overnight at 4 °C. Next day, the Alexa Fluor 568 donkey anti-Rabbit IgG (H+L) (ThermoFisher Scientific, A10042) secondary antibody was incubated for 60 min and followed with DAPI staining. An Olympus light microscope BX53 system was applied for images capture.

### Flow cytometry

The MOVAS cells (about 2–5 × 10^6^ cells) were collected by trypsin digestion, and these cells were washed with phosphate-buffered saline (PBS) for twice, and then centrifuged for 10 min in order to harvest cells. After re-suspending the cells with 500 μL PBS, 5 ml of pre-cold 70% ethanol was added to fix cells overnight at 4 °C. Next day, the cells were washed with PBS for twice after discarding ethanol. Then, 0.3 mg of Ribonuclease A (R5125; Sigma-Aldrich) and 0.015 mg of PI (P4864; Sigma-Aldrich) were applied to stain cells for 2 h in darkness. The stained cells were sorted by using a BD FACS Aria™ III sorter for cell cycle analysis. For apoptosis assay, the PE Annexin V Apoptosis Detection Kit I (559763, BD Pharmingen^TM^) was used, and the BD FACS Aria™ III sorter was applied to sorting cells.

### Transmission electron microscope

After being treated with indicated lenti-virus or UNC1999, the MOVAS cells were washed with PBS for twice. Cold 2.5% of glutaraldehyde was used to fix cells for 30 min, then, the fixed cells were scraped and collected by centrifugation. The cells were dehydrated by ethanol and acetone after further fixed in 2.5% of glutaraldehyde overnight at 4 °C. Dehydrated cells were soaked in resin epoxy and then embedded. Sixty-nanometer-thick slices were obtained for uranyl acetate and lead citrate staining. The images were got by using a transmission electron microscope (Hitachi HT7700).

### Statistical analysis

All the data were represented as mean ± standard deviation (SD) in the present study. Student’s two-tailed *t*-test was used to compare the means of two groups. Multiple groups comparisons were achieved by using one-way ANOVA tests with least significant difference (equal variances assumed) or Tamhane T2 (equal variances not assumed) in SPSS software (version 13.0). *p < *0.05 is considered as statistical significance.

## Electronic supplementary material

Supplemental materials

## References

[1] Erbel R (2014). 2014 ESC guidelines on the diagnosis and treatment of aortic diseases: document covering acute and chronic aortic diseases of the thoracic and abdominal aorta of the adult. The Task Force for the Diagnosis and Treatment of Aortic Diseases of the European Society of Cardiology (ESC). Eur. Heart J..

[2] Zhu L (2006). Mutations in myosin heavy chain 11 cause a syndrome associating thoracic aortic aneurysm/aortic dissection and patent ductus arteriosus. Nat. Genet..

[3] Larson EW, Edwards WD (1984). Risk factors for aortic dissection: a necropsy study of 161 cases. Am. J. Cardiol..

[4] Jiang DS, Yi X, Zhu XH, Wei X (2016). Experimental in vivo and ex vivo models for the study of human aortic dissection: promises and challenges. Am. J. Transl. Res..

[5] Huang J (2015). Myocardin is required for maintenance of vascular and visceral smooth muscle homeostasis during postnatal development. Proc. Natl. Acad. Sci. USA.

[6] Wang Y (2016). Dynamic autophagic activity affected the development of thoracic aortic dissection by regulating functional properties of smooth muscle cells. Biochem. Biophys. Res. Commun..

[7] Jia LX (2015). Mechanical stretch-induced endoplasmic reticulum stress, apoptosis and inflammation contribute to thoracic aortic aneurysm and dissection. J. Pathol..

[8] Liao WL (2015). Brahma-related gene 1 inhibits proliferation and migration of human aortic smooth muscle cells by directly up-regulating Ras-related associated with diabetes in the pathophysiologic processes of aortic dissection. J. Thorac. Cardiovasc. Surg..

[9] Mizushima N (1998). A protein conjugation system essential for autophagy. Nature.

[10] Galluzzi L (2012). Molecular definitions of cell death subroutines: recommendations of the Nomenclature Committee on Cell Death 2012. Cell Death Differ..

[11] Ding J (2013). The histone H3 methyltransferase G9A epigenetically activates the serine-glycine synthesis pathway to sustain cancer cell survival and proliferation. Cell Metab..

[12] Collins PL, Oltz EM (2013). Histone methylation keeps the brakes on autophagy. Mol. Cell Biol..

[13] Yuan Y (2013). Gossypol and an HMT G9a inhibitor act in synergy to induce cell death in pancreatic cancer cells. Cell Death Dis..

[14] Ciechomska IA, Przanowski P, Jackl J, Wojtas B, Kaminska B (2016). BIX01294, an inhibitor of histone methyltransferase, induces autophagy-dependent differentiation of glioma stem-like cells. Sci. Rep..

[15] Mitic T (2015). EZH2 modulates angiogenesis in vitro and in a mouse model of limb ischemia. Mol. Ther..

[16] Delgado-Olguin P (2014). Ezh2-mediated repression of a transcriptional pathway upstream of Mmp9 maintains integrity of the developing vasculature. Development.

[17] Aljubran SA (2012). Enhancer of zeste homolog 2 induces pulmonary artery smooth muscle cell proliferation. PLoS ONE.

[18] Huang Z (2017). MicroRNA-21 protects against cardiac hypoxia/reoxygenation injury by inhibiting excessive autophagy in H9c2 cells via the Akt/mTOR pathway. J. Cell Mol. Med..

[19] Periyasamy-Thandavan S, Jiang M, Schoenlein P, Dong Z (2009). Autophagy: molecular machinery, regulation, and implications for renal pathophysiology. Am. J. Physiol. Ren. Physiol..

[20] Corcelle E (2007). Control of the autophagy maturation step by the MAPK ERK and p38: lessons from environmental carcinogens. Autophagy.

[21] Gonzalez ME (2011). Histone methyltransferase EZH2 induces Akt-dependent genomic instability and BRCA1 inhibition in breast cancer. Cancer Res..

[22] Nienaber CA (2016). Aortic dissection. Nat. Rev. Dis. Prim..

[23] Baehrecke EH (2002). How death shapes life during development. Nat. Rev. Mol. Cell. Biol..

[24] Kwartler CS (2014). Overexpression of smooth muscle myosin heavy chain leads to activation of the unfolded protein response and autophagic turnover of thick filament-associated proteins in vascular smooth muscle cells. J. Biol. Chem..

[25] Zhi X., Feng W., Rong Y. & Liu R. Anatomy of autophagy: from the beginning to the end. *Cell Mol. Life Sci*. Epub ahead of print (2017) 10.1007/s00018-017-2657-z. 10.1007/s00018-017-2657-z PMC11105611 28939950

[26] Shintani T, Klionsky DJ (2004). Autophagy in health and disease: a double-edged sword. Science.

[27] Shimizu S, Yoshida T, Tsujioka M, Arakawa S (2014). Autophagic cell death and cancer. Int. J. Mol. Sci..

[28] Nassif M (2014). Pathogenic role of BECN1/Beclin 1 in the development of amyotrophic lateral sclerosis. Autophagy.

[29] Wang M (2016). Silibinin prevents autophagic cell death upon oxidative stress in cortical neurons and cerebral ischemia-reperfusion injury. Mol. Neurobiol..

[30] Matsui Y (2007). Distinct roles of autophagy in the heart during ischemia and reperfusion: roles of AMP-activated protein kinase and Beclin 1 in mediating autophagy. Circ. Res..

[31] Sadoshima J (2008). The role of autophagy during ischemia/reperfusion. Autophagy.

[32] Valentim L (2006). Urocortin inhibits Beclin1-mediated autophagic cell death in cardiac myocytes exposed to ischaemia/reperfusion injury. J. Mol. Cell. Cardiol..

[33] Knaapen MW (2001). Apoptotic versus autophagic cell death in heart failure. Cardiovasc. Res..

[34] Hein S (2003). Progression from compensated hypertrophy to failure in the pressure-overloaded human heart: structural deterioration and compensatory mechanisms. Circulation.

[35] Chang LC (2014). YC-1 inhibits proliferation of breast cancer cells by down-regulating EZH2 expression via activation of c-Cbl and ERK. Br. J. Pharmacol..

[36] Martinez-Lopez N, Athonvarangkul D, Mishall P, Sahu S, Singh R (2013). Autophagy proteins regulate ERK phosphorylation. Nat. Commun..

[37] Wei PF (2015). Differential ERK activation during autophagy induced by europium hydroxide nanorods and trehalose: maximum clearance of huntingtin aggregates through combined treatment. Biomaterials.

[38] Xiao Y (2016). Activation of ERK1/2 ameliorates liver steatosis in leptin receptor-deficient (db/db) mice via stimulating ATG7-dependent autophagy. Diabetes.

[39] Feng J, Ge S, Zhang L, Che H, Liang C (2016). Aortic dissection is associated with reduced polycystin-1 expression, an abnormality that leads to increased ERK phosphorylation in vascular smooth muscle cells. Eur. J. Histochem..

[40] Zhang W, Shu C, Li Q, Li M, Li X (2015). Adiponectin affects vascular smooth muscle cell proliferation and apoptosis through modulation of the mitofusin-2-mediated Ras-Raf-Erk1/2 signaling pathway. Mol. Med. Rep..

[41] Liu B (2011). Acetylbritannilactone induces G1 arrest and apoptosis in vascular smooth muscle cells. Int. J. Cardiol..

[42] Wang Z (2014). Angiotensin-II induces phosphorylation of ERK1/2 and promotes aortic adventitial fibroblasts differentiating into myofibroblasts during aortic dissection formation. J. Mol. Histol..

[43] Chen, K. et al. Alternative splicing of EZH2 pre-mRNA by SF3B3 contributes to the tumorigenic potential of renal cancer. *Clin. Cancer Res*. **23**, 3428-3441 (2016). 10.1158/1078-0432.CCR-16-2020 PMC5440213 27879367

[44] Jiang DS (2017). Aberrant epicardial adipose tissue extracellular matrix remodeling in patients with severe ischemic cardiomyopathy: insight from comparative quantitative proteomics. Sci. Rep..

[45] Jiang DS (2016). The potential role of lysosome-associated membrane protein 3 (LAMP3) on cardiac remodelling. Am. J. Transl. Res.

[46] Chang E (2017). Synergistic inhibition of glioma cell proliferation by Withaferin A and tumor treating fields. J. Neurooncol..

[47] Jiang DS (2013). Role of interferon regulatory factor 4 in the regulation of pathological cardiac hypertrophy. Hypertension.

[48] Jiang DS (2014). Interferon regulatory factor 1 is required for cardiac remodeling in response to pressure overload. Hypertension.

[49] Jiang DS (2014). Interferon regulatory factor 7 functions as a novel negative regulator of pathological cardiac hypertrophy. Hypertension.

[50] Jiang DS (2014). Interferon regulatory factor 9 protects against cardiac hypertrophy by targeting myocardin. Hypertension.

[51] Jiang DS (2014). IRF8 suppresses pathological cardiac remodelling by inhibiting calcineurin signalling. Nat. Commun..

[52] Jiang DS (2014). Signal regulatory protein-alpha protects against cardiac hypertrophy via the disruption of toll-like receptor 4 signaling. Hypertension.

