# Supplementary material for: EZH2 inhibits autophagic cell death of aortic vascular smooth muscle cells to affect aortic dissection
Source: Cell Death Dis. 2018 Feb 7;9(2):180. doi: 10.1038/s41419-017-0213-2 (PMC5833461; doi:10.1038/s41419-017-0213-2)
Supplement: Supplementary file 1 — Supplemental materials [file 41419_2017_213_MOESM1_ESM.docx]

**Supplemental Tables and Figures**

**Table S1. The clinical information of patients with AD and NAD**

| **Clinical Indicators** | **AD (n=16)** | **NAD (n=15)** | |
| --- | --- | --- | --- |
|  |  | **DCM (n=5)**  **/CAD (n=3)** | **Donor (n=7)** |
| Sex (M/F) | 12/4 | 7/1 | 6/1 |
| Age (year) | 45.75±2.13 | 49.5±6.5 | 29.57±2.56 |
| BMI (kg/m^2^) | 26.32±0.88 | 23.83±1.31 | 23.72±0.52 |
| Smokers (n, %) | 6 (37.5%) | 1 (12.5%) | 1 (14.29%) |
| Diabetes (n, %) | 2 (12.5%) | 1 (12.5%) | 0 (0%) |
| Hypertension (n, %) | 12 (75%) | 2 (25%) | 0 (0%) |
| Aortic Diameter (mm) | 46.00±3.38 | N/A | N/A |
| EF (%) | 58.33±1.53 | 30.57±4.25 | 61.29±0.68 |
| D-dimer (ug/L) | 579.36 (1.73, 5039) | N/A | N/A |

AD: aortic dissection; NAD: non-aortic dissection; DCM: dilated cardiomyopathy; CAD: coronary artery disease; BMI: body mass index; EF: ejection fraction


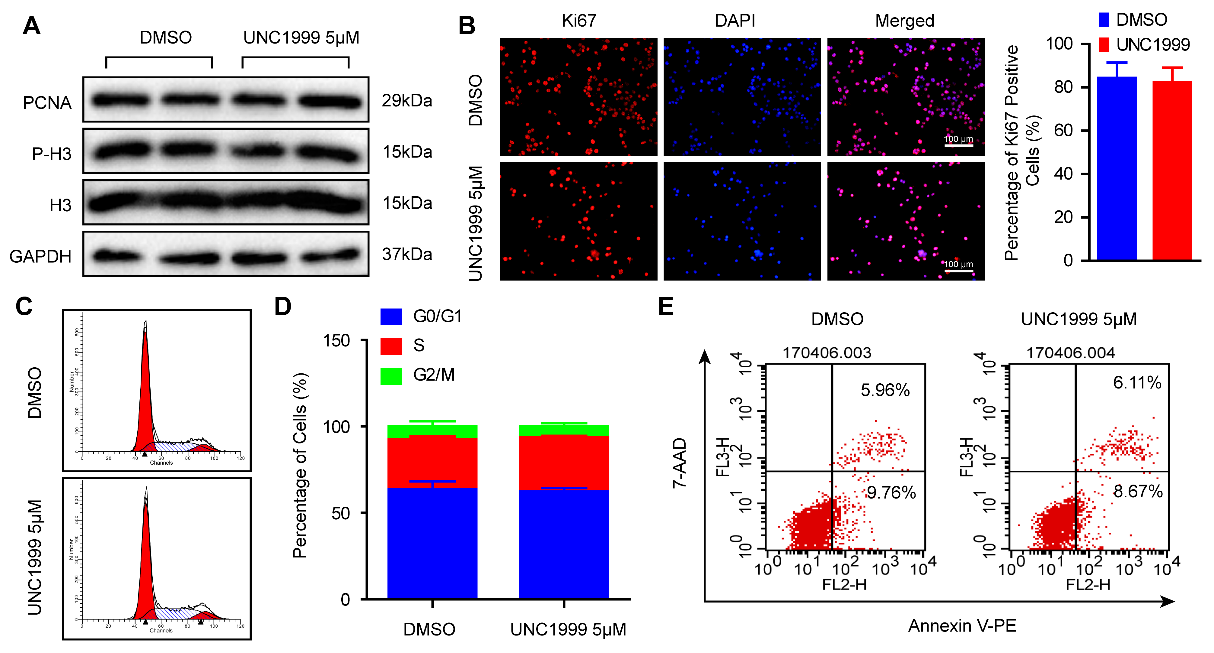


**Figure S1. EZH2 inhibition by UNC1999 has no effects on VSMCs proliferation and apoptosis.** **A,** Representative western blots of proliferation markers PCNA and phosphorylation H3 (p-H3) in VSMCs after DMSO or 5 μM of UNC1999 stimulation (n=4). **B,** Representative images and quantitative results of Ki67 immunofluorescence staining, red: Ki67, blue, nucleus (scale bar, 100 μm, n=3). **C and D,** Representative images of cell cycle detected by flow cytometry (C), the statistical results of cell ratio at different cell phases (D) (n=3). **E,** Representative images of cell apoptosis evaluated by flow cytometry (n=3). UNC1999 treatment 48h for all these experiments.


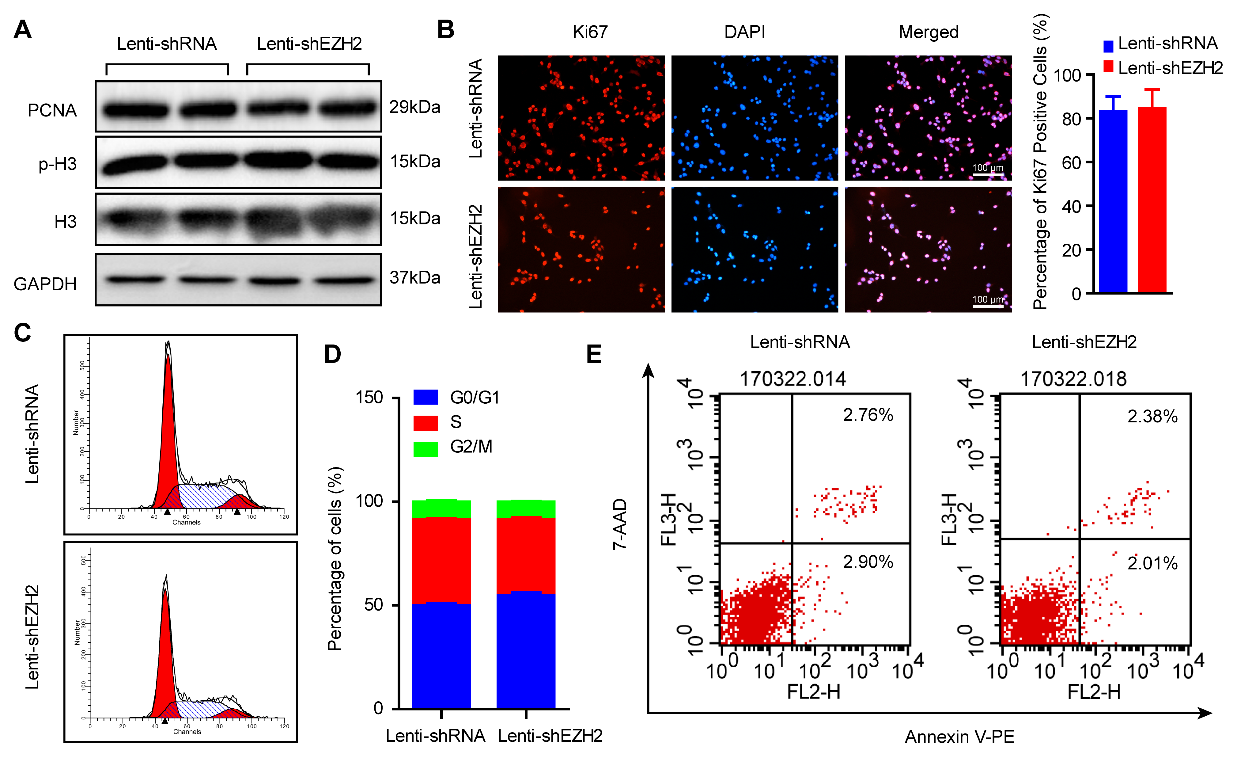


**Figure S2. VSMCs proliferation and apoptosis do not affected by knockdown of EZH2.** **A,** The PCNA, p-H3 protein level was detected by western blot in VSMCs (n=4). **B,** The Ki67 protein level was verified by immunofluorescence staining in VSMCs, red: Ki67, blue, nucleus (scale bar, 100 μm, n=3). **C and D,** Cell cycle was measured by flow cytometry, **C,** Representative results; **D,** the statistical results of cell ratio at different cell phases (n=3). **E,** VSMCs apoptosis was evaluated by PE Annexin V Apoptosis Detection Kit and detected by flow cytometry (n=3).


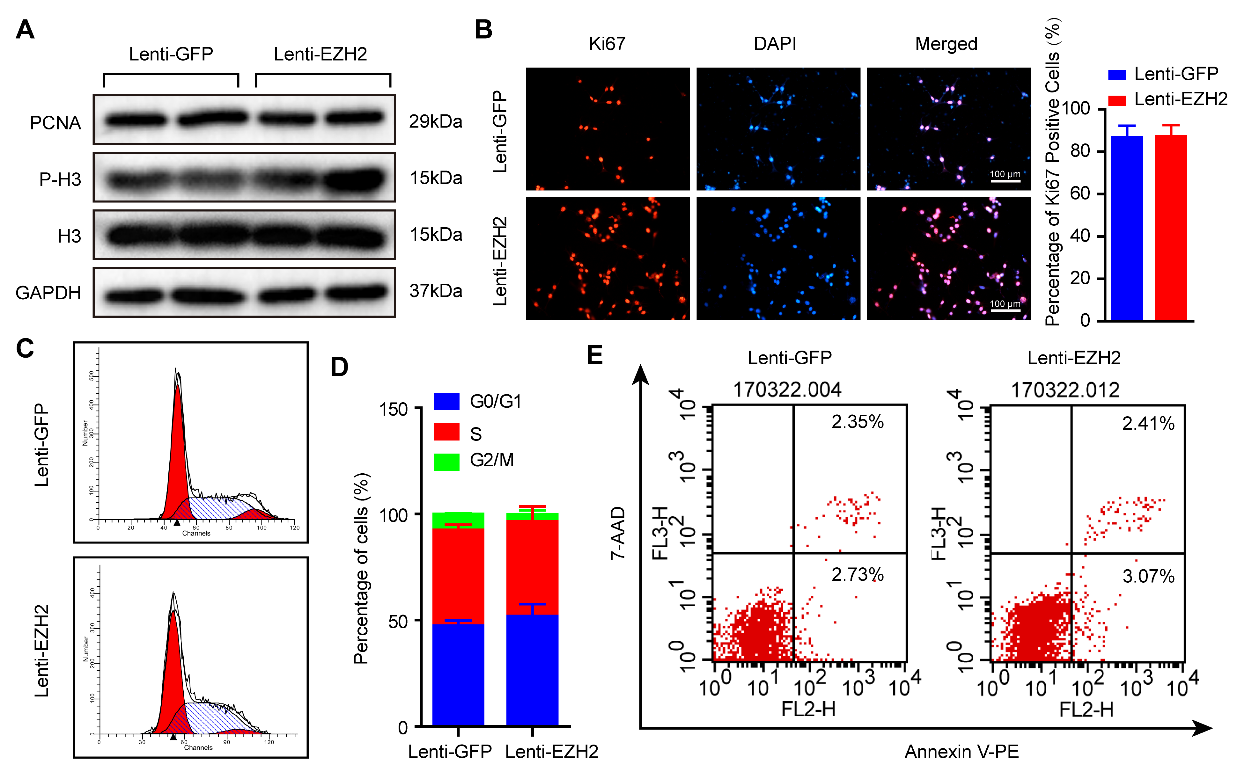


**Figure S3. Neither VSMCs proliferation nor apoptosis regulated by EZH2 overexpression.** **A,** The western blots of proliferation markers PCNA and p-H3 in VSMCs with GFP or EZH2 overexpression (n=4). **B,** Immunofluorescence staining was performed to evaluate Ki67 protein level in VSMCs, red: Ki67, blue, nucleus (scale bar, 100 μm, n=3). **C and D,** Flow cytometry was performed to detect cell cycle of VSMCs. **C,** Representative images of flow cytometry; **D,** the statistical results of cell ratio at different cell phases (n=3). **E,** Representative images of VSMCs apoptosis measured by flow cytometry (n=3).


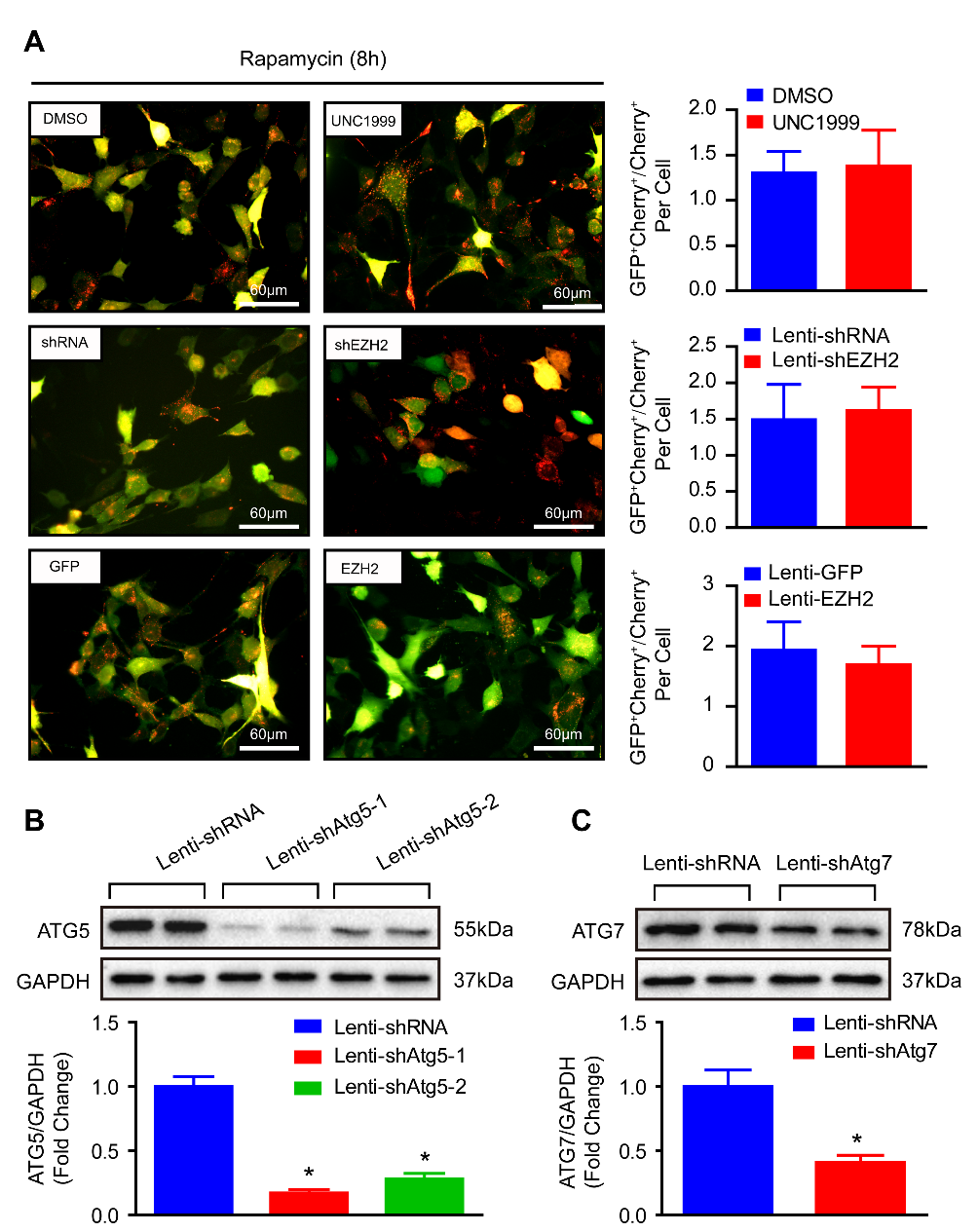


**Figure S4. Identification of ATG5 and ATG7 knockdown.** **A,** The mCherry-GFP-LC3 was overexpressed in the indicated VSMCs, which were subsequently stimulated with rapamycin (150 nM) for 8 hours. Yellow and red indicate autophagosomes or autolysosomes, respectively (scale bar, 60 μm, n=3). Right panel, the ratio of autophagosomes to autolysosomes. **B,** Representative western blots and statistical results of ATG5 (n=4). **C,** ATG7 protein level was detected by western blot (n=4). GAPDH serves as loading control. **p<*0.05 vs. lenti-shRNA.


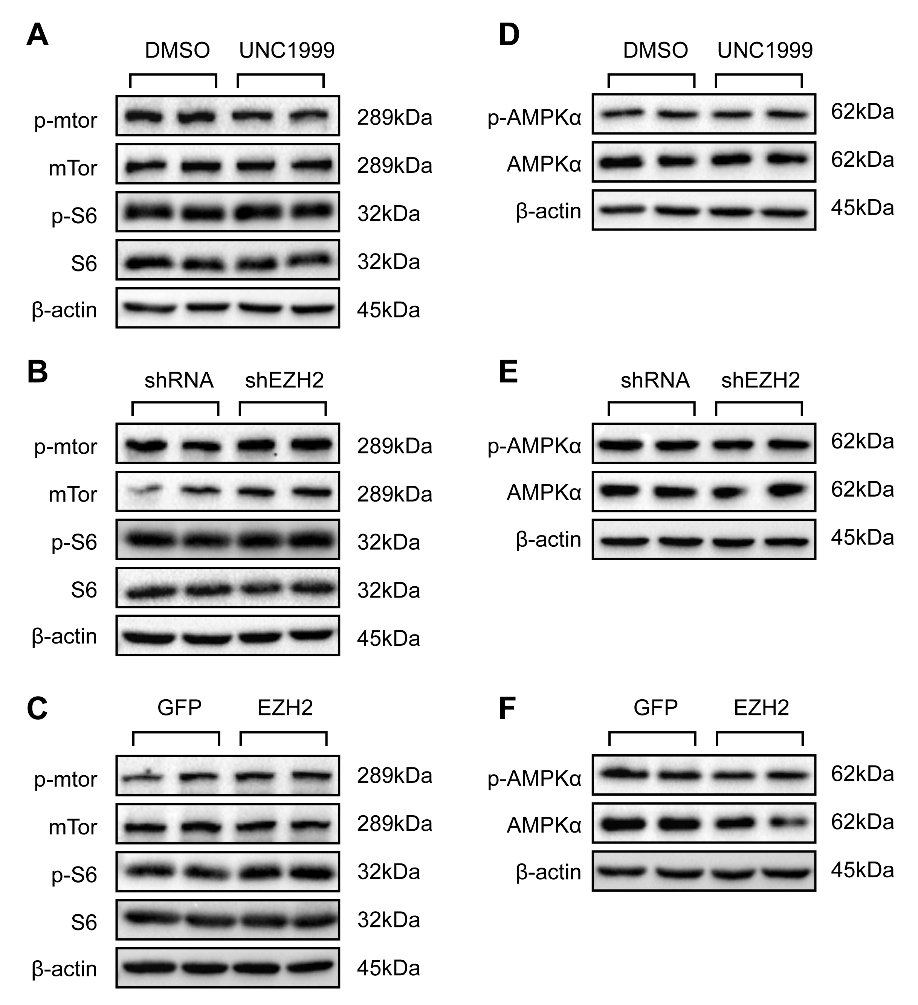


**Figure S5. The mTOR and AMPKα signaling pathway were independent of the role of on EZH2 autophagy. A-C,** The phosphorylation levels of mTOR, S6 in the VSMCs treated with UNC1999 for 24h (A), lenti-shEZH2 (B), or lenti-EZH2 (C). **D-F,** The phosphorylation levels of AMPKα in the VSMCs treated with UNC1999 (D), lenti-shEZH2 (E), or lenti-EZH2 (F). β-actin serves as loading control.


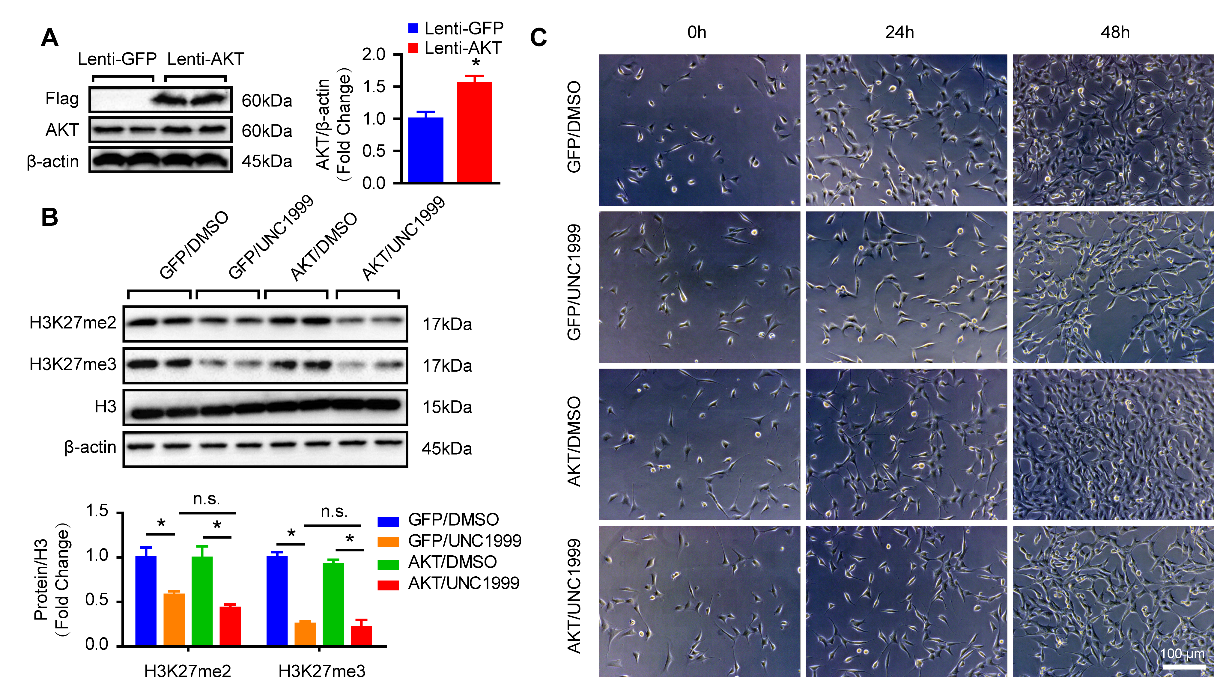


**Figure S6. AKT signaling did not mediate the role of EZH2 on apoptotic cell death of VSMCs. A,** The protein levels of Flag, AKT in VSMCs infected with lenti-GFP or lenti-AKT were detected by western blot (n=4). **B,** The methylation level of H3K27me2, H3K27me3 in AKT overexpressed VSMCs treated with or without UNC1999 for 24h (n=4), **p*<0.05. **C,** The representative VSMCs images under light microscopy after treated with indicated stimulus. β-actin serves as loading control.


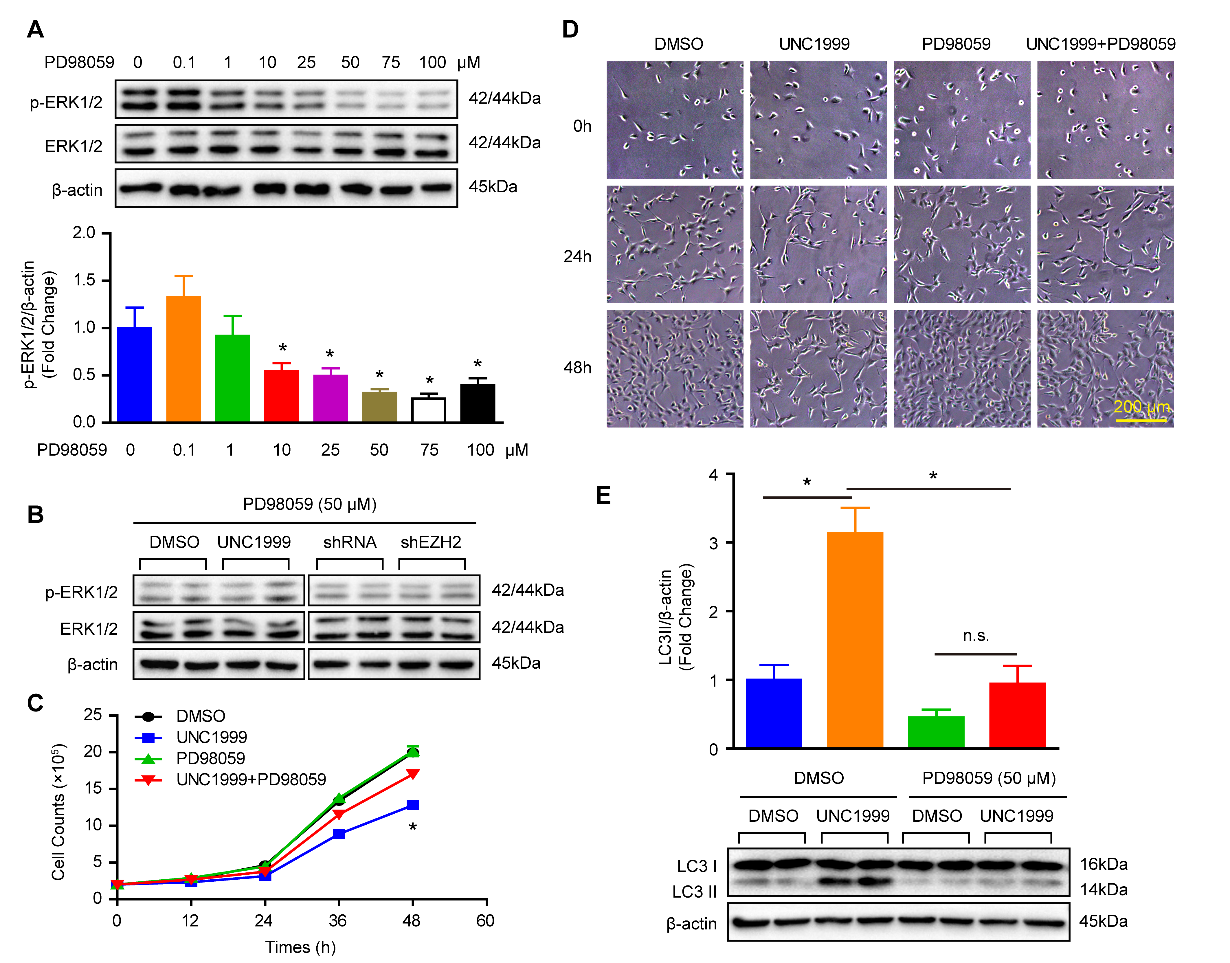


**Figure S7. Inhibition of MEK-ERK1/2 signaling pathway largely rescued the apoptotic cell death of VSMCs. A,** The phosphorylation level of ERK1/2 in the VSMCs treated with different concentration of PD98059 (a MEK1 inhibitor) for 2h (n=4), **p*<0.05 vs. 0 μM of PD98059. **B,** The phosphorylation level of ERK1/2 in VSMCs treated with both UNC1999 and PD98059, or lenti-shEZH2 and PD98059 for 6h (n=4). **C,** The growth curve of VSMCs treated with indicated stimulus (n=3, **p*<0.05 vs UNC1999+PD98059). **D,** The representative VSMCs images under light microscopy after treated with indicated stimulus. **E,** The protein levels of LC3II/I was detected by using western blot in VSMCs treated with indicated stimulus for 6h (n=4). β-actin serves as loading control. **p*<0.05.
